# Development of a Human Factors–Based Guideline to Support the Design, Evaluation, and Continuous Improvement of Clinical Decision Support

**DOI:** 10.1016/j.mcpdig.2024.11.003

**Published:** 2024-11-27

**Authors:** Selvana Awad, Thomas Loveday, Richard Lau, Melissa T. Baysari

**Affiliations:** a Biomedical Informatics and Digital Health, School of Medical Sciences, Faculty of Medicine and Health, The University of Sydney, Australia; b eHealth NSW, New South Wales, Australia

## Abstract

**Objective:**

To develop a vendor-agnostic, human factors (HF)-based guideline to guide the design, evaluation, and continuous improvement of clinical decision support (CDS).

**Participants and Methods:**

The study used a 2-phased iterative approach between June 2022 and June 2024. Phase 1 involved a search for relevant industry standards and literature and consultation with multidisciplinary subject matter experts. Phase 2 involved a workshop with 30 health care and academic stakeholders to evaluate face validity and perceived usefulness of the initial section of the guideline. Participants were asked if the guideline met their expectations, to report on usefulness and ease of use and to suggest areas for improvement.

**Results:**

Phase 1 resulted in a compilation of accessible, best practice, and context-appropriate HF guidance for CDS design and optimization. The guideline supports users in determining whether use of CDS is appropriate, and if yes, CDS options and design guidance. During phase 2, the guideline addressed 15 of participants’ 19 expectations for a CDS guideline. Participants said the guideline was helpful, comprehensive, easy to use, and provided step-by-step guidance, boundaries, and transparency around CDS decisions. Participants recommended strengthening guidance around the need to understand system capabilities and the technical burden or complexity of CDS, and further guidance on how to approach CDS optimization using the guideline.

**Conclusion:**

The 2-phased iterative development and feedback process resulted in the development of an HF-informed guideline to provide consolidated, accessible, and current best practice guidance on the appropriateness of CDS and CDS options, as well as designing, evaluating, and continuously improving CDS. Future work will evaluate the impact and implementation of the guideline in real-world settings.

Clinical decision support (CDS) embedded within clinical information systems provides health care workers and patients with relevant knowledge, contextual, and person-specific information, at appropriate times to support the delivery of safe, quality patient care.^1^, ^2^, ^3^ Overreliance on CDS functionality to improve practice and/or prevent errors may result in unintended consequences.^4^^,^^5^ Implementing excessive or inappropriate forms of CDS to prevent all possible errors may reduce usability, for example, by triggering too many alerts, cluttering the workflow and/or slowing down the system.^4^ Clinical decision support can also adversely affect clinical decision making by introducing cognitive biases such as anchoring, which may lead to diagnostic errors.^6^ Poor CDS usability can result in inefficiencies, increased cognitive effort, downstream costs, staff burnout, workarounds, and patient harm.^6^, ^7^, ^8^, ^9^, ^10^, ^11^, ^12^, ^13^ Ultimately, poorly designed or inappropriate CDS can lead to low adoption by users so that any potential benefits intended by the CDS are not realized.^14^^,^^15^

Within NSW in Australia, interruptive alerting is a commonly considered form of CDS to address clinical problems (eg, when developing recommendations in response to patient safety incidents) and alert fatigue has been reported as a clinical risk. Alert fatigue occurs when excessive and inappropriate alerts desensitize users, leading them to override both clinically relevant and irrelevant alerts.^16^, ^17^, ^18^ Interruptive forms of CDS such as alerts may provide benefits when integrated in the workflow, are implemented judiciously by reserving them for high risk or safety-critical situations requiring immediate action, and are sufficiently specific and sensitive.^2^^,^^17^^,^^19^, ^20^, ^21^, ^22^ Failure to consider the broader ecosystem, the presence of other alert triggers, the relative priority of the alert under consideration, and the impact of implemented alerts may lead to decreased effectiveness and contribute to alert fatigue.^5^^,^^17^^,^^23^, ^24^, ^25^

In addition to alerts, there is growing recognition that a diverse range of CDS modalities/tools is available to enhance decision-making in the clinical workflow.^1^^,^^6^ “Passive” CDS mechanisms can make it easier for clinicians to do the right thing without interrupting their workflows and serve as nudge interventions, which guide clinicians toward making appropriate decisions through system design.^15^^,^^26^ To ensure effective utilization and uptake, research has shown that CDS should be integrated into the clinical workflow to support the cognitive process of decision making and ensure consideration of the wider context in which it is being used.^27^, ^28^, ^29^, ^30^ To achieve this, it is essential to carefully consider the most appropriate types or forms of CDS for the specific use case.^2^

Human factors (HF) (or ergonomics) is a science-based discipline that explores the interaction between humans and systems by understanding factors that facilitate the completion of work.^31^ It involves the application of theory, principles, data, and methods to optimize human wellbeing and overall system performance^32^ and is increasingly being used to inform the design and redesign of health information technologies (HITs).^33^ This is because HF provides an effective, systems-based approach for understanding the relationships between system elements and components, and therefore optimal ways for designing safe complex systems.^34^^,^^35^

Established HF-based standards for decision support such as alerts/alarms have been widely adopted across other industries such as process control, manufacturing, and aviation.^33^^,^^36^, ^37^, ^38^, ^39^, ^40^ However, limited consolidated guidance or formal standards on the basis of HF and safety engineering are currently available in the HIT context to support the design of CDS. Although some guidance exists around optimal CDS design, including design checklists,^20^^,^^33^^,^^41^, ^42^, ^43^, ^44^, ^45^, ^46^, ^47^ these are spread across multiple sources, rarely delve into the appropriateness of CDS as a solution, tend to focus on interface design, and have not systematically leveraged HF-based standards from other sectors. Furthermore, their practical utility, usability, and application to inform decision making about CDS in practice are unclear.^33^^,^^41^ The CDS implementation guide by Osheroff et al^2^ offers a comprehensive range of information about optimal design and implementation of CDS. However, it has not been updated since 2012, and its content may not be accessible to system designers owing to its length and cost.

Thus, within our organization, which is responsible for digitally enabling hospitals across NSW in Australia, we recognized the need for developing consolidated, accessible, comprehensive and up-to-date HF-informed guidance on the selection and design of CDS. We envisioned our guideline to be used by both experienced and inexperienced CDS designers/implementers to improve consistency in the approach taken toward appropriate CDS inclusion and design and support conversations where CDS is being requested but may not be appropriate. Our goal was to synthesize HF knowledge from available literature and health and nonhealth industry standards to create an accessible, practical guideline on CDS selection and design.

### Objectives

This project aimed to develop a vendor-agnostic, HF-based guideline to guide the design, evaluation, and continuous improvement of CDS.

## Particpants and Methods

**Figure 1:**
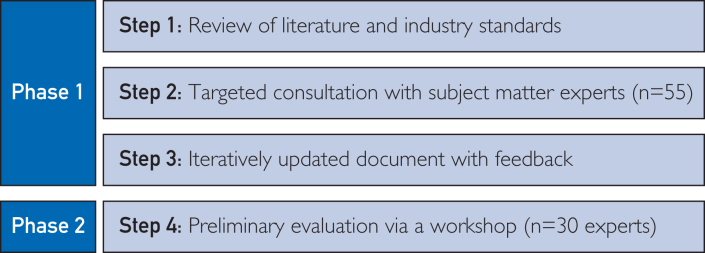
Two-phased development and iterative feedback process.

Development of the CDS guideline consisted of a 2-phased iterative process involving an initial development and consultation phase, followed by a workshop-based preliminary evaluation. Figure 1 describes the 2-phase development process. This project was determined to be a quality improvement study, and ethics approval was not required.

### Phase 1: Initial Development and Consultation

The initial development phase involved a desktop exercise to identify relevant literature that could be used to inform the content. This included a literature search of databases including Medline (Ovid) and Embase (Ovid) using appropriate keywords to identify relevant articles on CDS (Appendix 1, available online at http://www.mcpiqojournal.org). Industry standards related to HF design and alarms management were also identified and reviewed.^9^^,^^36^^,^^37^^,^^40^^,^^48^, ^49^, ^50^, ^51^, ^52^, ^53^ These appear in Appendix 2 (available online at http://www.mcpiqojournal.org). Industry standards were considered relevant if they included guidance on interface design that could be applied to the CDS context and/or the design, testing, and continuous improvement process. These sources were reviewed by a clinical informatics professional with expertise in design, HF, and safety and quality, who extracted and synthesized relevant information to develop a first draft of the CDS guideline. Both theoretical, best practice knowledge (eg, articles containing design theory/principles or guidance on processes) and practical learnings (eg, where CDS tools were designed and evaluated) were incorporated into the first draft.

Consultation was conducted to seek input from subject matter experts and gather iterative feedback on the draft document. Experts were purposively selected to provide input and feedback on the basis of their roles, core expertise, and experience with design of CDS. Input from subject matter experts was provided through short meetings (n=3) and/or written feedback (n=15) in the document itself, depending on the preference of the expert. After consultation with individual experts, the draft guideline was also shared with a multidisciplinary digital health safety and quality governance committee for review. Overall, 15 individual subject matter experts with varying expertise, and a safety and quality governance committee (Table 1), provided input. The guideline was updated and refined on the basis of feedback received.

**Table 1:** Core Expertise of Subject Matter Experts Who Contributed to and/or Reviewed the Draft Clinical Decision Support Guideline as Part of Phase 1

Core expertise	Number
Pharmacy informatics	3
Nursing informatics	2
Medical informatics	3
Human factors	5
Human-centered design	1
Health information technology (HIT) delivery	1
Multidisciplinary statewide digital health safety and quality governance group, covering policy, clinical, patient safety/clinical governance, digital health, and consumer perspectives	Approximately 30 representatives
Total	55

### Phase 2: Preliminary Evaluation via a Workshop

After phase 1, a workshop was held with health care and academic stakeholders to evaluate face validity and perceived usefulness of a key section of the guideline, which includes a flowchart and table to guide decision making about the appropriateness of CDS and potential options (Appendices 2 and 3, available online at http://www.mcpiqojournal.org).

The workshop was convened during an international HF conference (Context Sensitive Health Informatics 2023, Sydney, Australia) and was open to all conference delegates to take part (capped at 30 participants, on a first-come first-served basis). In total, 30 participants with backgrounds in health care, including clinical and non-clinical roles (n=16), research or academia (n=12), and other areas (n=3), such as informatics or clinical governance, participated in the workshop. One participant had both a health care and research/academia background. At the start of the workshop, a real-time collaborative web platform (Padlet^54^) was used to collect data about participants’ core work, the reasons they joined the workshop, whether they have worked on designing and implementing CDS, and their CDS challenges and needs. Participants were also asked to comment on what they were looking for in a CDS guideline. This data was categorized into a series of expectations by 2 researchers (S.A., M.T.B.) using a general inductive approach. They then assessed whether the guideline fully addressed, partially addressed, or did not address each expectation.

During the workshop, participants were provided with an overview of the CDS guideline with a particular focus on the section being tested (Appendix 3a, b, available online at http://www.mcpiqojournal.org). The participants were then divided into small groups and provided with 2 case studies adapted from real CDS projects. One scenario covered blood product management and the other, pediatric medication prescription (Appendix 4, available online at http://www.mcpiqojournal.org). They were instructed to apply the CDS guideline to determine whether a CDS solution was appropriate for each case study, and if yes, which option/type of CDS would be beneficial. After small group discussions, a broader discussion took place where participants were asked to provide feedback on the CDS guideline using Padlet.^54^ In particular, participants were asked to provide feedback on what they learned about CDS options, the guideline’s overall usefulness, and share reflections on applying the guideline. Two researchers (S.A., M.T.B.) inductively coded participant responses into what worked well, opportunities for improvement, and learnings to support future application.

## Results

### Phase 1: Initial Development and Consultation

The first phase, which involved literature searches and targeted consultation, culminated in a draft CDS guideline, which includes 4 sections (Appendix 5, available online at http://www.mcpiqojournal.org).

**Figure 2:**
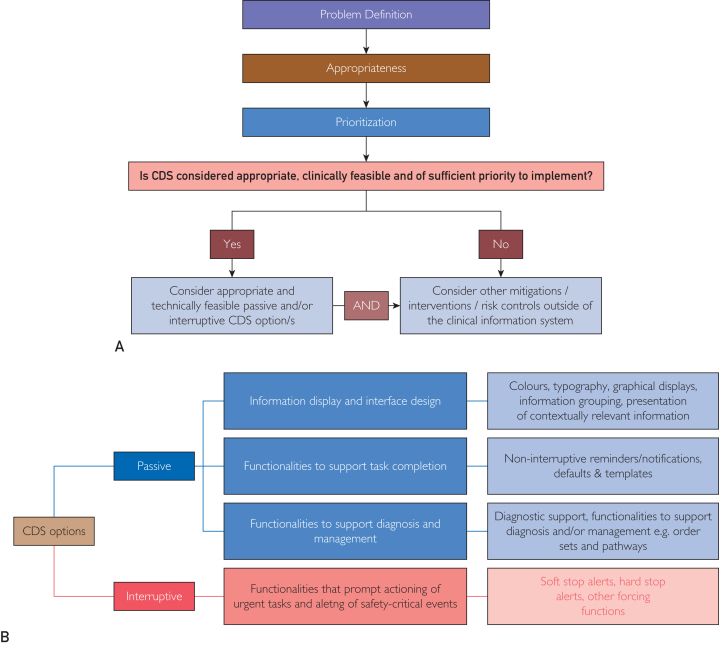
(A) Steps for determining whether clinical decision support (CDS) is appropriate (full details available in Appendix 3a, b, available online at http://www.mcpiqojournal.org). (B) CDS options that could be considered as outlined in the guideline (full details available in Appendix 3b, available online at http://www.mcpiqojournal.org).

Figure 2A, B summarizes the key flowchart and table in Section 2 of the guideline. Detailed versions can be found in Appendix 3a, b (available online at http://www.mcpiqojournal.org). Appendix 6 (available online at http://www.mcpiqojournal.org) summarizes sources used, which sections of the guideline they informed, and examples of content that were drawn from each source.

Consultation with subject matter experts resulted in modifications related to the feasibility of recommendations and the inclusion of content on the basis of lived experience around what designers of CDS require guidance on, pain points, and other relevant design considerations. For example, some prescriptive recommendations related to CDS types were reframed as design principles in response to feedback that it may not always be possible to comply with recommendations owing to system constraints. The flexible application of principles within system constraints was considered more feasible particularly where off-the-shelf solutions were being configured.

### Phase 2: Preliminary Evaluation via a Workshop

#### Expectations of a CDS Guideline

When asked about their CDS challenges and needs, and therefore expectations of a CDS guideline, participants listed a range of expectations including the need for guidance on the appropriateness of CDS and CDS types on the basis of the problem at hand, optimal design and evaluation of CDS, and implementation of CDS. Participants also provided comments on their expectations of the nature of the guideline, for example, the need for recommendations to be practical and clear. They expected the guideline to serve as an authoritative, evidence-based, risk-based, and practical guide to support conversations with clinicians and other stakeholders about the appropriateness of CDS and CDS options.

Table 2 provides a summary of participants’ expectations of a CDS guideline and an assessment of whether the CDS guideline at the time of the workshop met these expectations. Overall, 19 expectations were identified. When assessing whether the guideline included content that would meet participants’ expectations, the guideline fully addressed 15 of the 19 expectations, partially addressed 3 of the 19 expectations, and did not address of 1 of the 19 expectations (Table 2).

**Table 2:** Participants’ Expectations of a Clinical Decision Support (CDS) Guideline

Expectations	Does the CDS guideline provide guidance on this?
Appropriateness of CDS and CDS types	
Considerations before deciding to implement CDS	Yes
Guidance on whether CDS is an appropriate solution to address the underlying problem	Yes
A model risk assessment framework and guidance on what nature of CDS best suits each problem’s type	Yes
Understanding potential unintended downstream consequences of CDS	Partial
Estimating benefit and uptake in early discovery stages	Yes
Optimal design and evaluation of CDS	
Steps to guide the design process	Yes
Presenting complex information in a way that’s easy for users to understand without misinterpretation	Yes—section 4 (however this was not tested during phase 2)
Workflow integration of CDS including whether the CDS will target the right time in the decision-making process	Yes
Understanding potential impact on workflows/practices/culture/behaviors	Yes
Evidence-based design patterns and tools	Yes—section 4 (however, this was not tested during phase 2)
Prioritization, sensitivity, and precision of the CDS to ensure effectiveness without introducing fatigue	Yes
Optimal implementation of CDS	
Guidance on implementation within a hospital setting and potential barriers from operational staff for integration	No
Methodology with reference to change management principles	Partial
Guidance on embedding CDS into work processes	Partial
Nature of the guideline	
A guide that is practical and applicable, clear, and can support/integrate with other problem solving and improvement methodology	Yes
A model risk assessment framework	Yes
An authoritative source that can be used to develop a high-quality CDS with other team members who may have a limited understanding of the impacts of the introduction of CDS	Yes
Step-by-step: good guiding questions to make you reflect	Yes
Hands on, ready-to-use advice	Yes

#### What Worked Well

After application of the guideline, 10 of the 11 participants who responded to the question about the guideline’s usefulness indicated that the guideline was useful. They made comments about the guideline being helpful, comprehensive, easy to use, and providing step-by-step guidance, boundaries, and transparency around CDS decisions: *“logical, step-by-step guidance, comprehensive guidelines/instructions”*.

Participants also specifically valued the focus on what does not need a CDS solution, rather than assuming that a CDS solution would address the problem at hand: *“great with a focus on what does not need a CDS solution”* and *“useful to consider if CDS will actually fix the problem”*. In particular, participants found the guideline useful to *“guide discussion and get to the root issue”* and *“very helpful as a reflection for doing the assessment during the process and as an evaluation”*. Overall, participants expressed an openness and interest in applying the CDS guideline to relevant projects.

#### Opportunities for Improvement

When asked to provide feedback on how the guideline could be improved, participants recommended strengthening guidance around the need to understand system capabilities and the technical burden or complexity of CDS, which *“may be a huge determinant of the ability for it to be implemented and maintained and for its consequences”*. In addition, including guidance to prompt consideration of lower fidelity solutions was recommended: *“a fully functional CDS is not always the solution. Sometimes lower fidelity solutions are best. We too often jump to the most complex solution when simpler is better”.* Participants also recommended enhancing guidance around considering combinations of CDS options, which may be appropriate in some cases.

Participants recommended further guidance on how to approach CDS optimization using the guideline, how to convene an appropriate group to apply the guideline, and directing users to consider design patterns and relevant design guidance when considering how to design selected CDS option(s).

#### Learnings to Support Future Application of the Guideline

When asked to provide advice on future application of the guideline, feedback was provided around the people who should apply the guideline, the process of application, and supporting tools.

##### People

With respect to people applying the guideline, participants highlighted the importance of having multiple perspectives and the appropriate personnel involved in discussions to ensure that required information such as an understanding of the workflow and technically feasible options are available to inform decisions: *“…including clinicians and people who know the workflows are really helpful…combined with others who know CDS and what is technically feasible.”*

##### Processes

Participants recommended that application of the guideline should occur as an interactive and collaborative group discussion and allow adequate time for discussion: *“useful to work through the guideline as an interactive group discussion”.*

A suggestion was also made to *“have a team with the context and/or pre-work map current processes/workarounds/policies to inform the use of the framework”* before discussions. Finally, a recommendation was made for the guideline to be applied iteratively with *“CDS design seen as an ongoing endeavor”* and *“need to talk, trial and test. And then repeat the process”.*

##### Tools

With respect to supporting tools, suggestions were made to *“build the framework into current decision/problem solving tools used and make available templates for each step, for example, similar to value proposition templates”.*

## Discussion

This study described a two-phased iterative development and feedback process of a HF-informed best practice CDS guideline. Phase 1 resulted in development of a 4-section, HF-informed best practice guideline. While established standards on alarm systems and similar are widely adopted across other industries, equivalent standards are lacking in the CDS context. Although some aspects of these industry standards are not directly applicable to CDS, many best practice principles were found to be translatable to the CDS context. CDS is often designed and implemented within the context of time and resource-constrained digital health projects. As such, CDS design decisions may need to occur rapidly and designers of CDS systems may not have the opportunity to access guidance in disparate sources, including industry standards that may be difficult for non-experts to consume. Consultation with subject matter experts during phase 1 proved highly valuable. A key outcome of this being the reframing of particular recommendations as principles.

Feedback from workshop participants revealed that the guideline was a helpful tool in providing structure around CDS discussions, and many participants expressed an interest in applying it in the future. The guideline’s prompting around understanding factors contributing to the problem on the basis of a systems thinking approach, and therefore whether CDS is an appropriate solution, was viewed as particularly helpful. The concept of understanding the problem and key contributing factors to design appropriate interventions/solutions is used in other industries and not new in the health care context (eg, to support quality improvement initiatives and incident investigations). However, it is rarely used to inform decision making about CDS.^55^^,^^56^ As we know from our context, decisions about introducing CDS, for example in response to a patient safety event, tend to be undertaken from a default position that CDS, usually in the form of interruptive alerts, will resolve the issue.^57^ Without appropriate rationalization of the CDS to ensure it will solve the problem and is appropriate within the broader ecosystem, the CDS may result in unintended consequences.^5^^,^^42^^,^^43^

With respect to improvement opportunities, participants recommended including greater emphasis on considering the technical feasibility of CDS solutions, which is a significant determining factor when making decisions about CDS. This is consistent with other literature, which highlights the importance of weighing up the technical complexity, feasibility and maintenance burden of CDS, with potential clinical benefits.^2^^,^^6^^,^^58^

Although guidance exists on what constitutes quality evidence-based clinical practice guidelines, little information exists on what should be included in HIT design guidelines to make them useful.^59^ This study generated novel learnings about what potential users of CDS design guidelines consider useful for inclusion to guide design decision making. When asked to articulate their expectations of a CDS guideline on the basis of CDS needs and challenges faced, workshop participants expected guidance on (1) when it is appropriate to consider CDS; (2) CDS options that may be suitable depending on the problem at hand; and (3) the optimal design, implementation, and evaluation of CDS. In particular, participants requested an evidence-informed guide to provide structure and process around CDS discussions and decision making. This is likely because without effective CDS governance practices and processes, CDS discussions can be unstructured and opinion based and lead to suboptimal decision making.^43^

Although guidelines can be helpful summaries of available evidence and best practice, their utility can only be actualized if they are usable and are practically applied to real-world problems. The workshop generated valuable findings with respect to ideas that would enhance future application of the guideline. In particular, participants recommended the need for CDS discussions to include multidisciplinary representation that includes both clinical and technical expertise, which is consistent with other recommendations about CDS governance.^60^

Research around HF-based CDS design recommends that the design of CDS should be a continuous process that takes into account the broader work context and entire sociotechnical system.^25^^,^^28^^,^^61^^,^^62^ During the workshop, participants highlighted the importance of applying the guideline iteratively to ensure that CDS is continuously improved and where required, decommissioned. This is an important finding as research demonstrates that a common challenge with CDS is that it is not frequently reviewed and where required, decommissioned, which leads to excessive or inappropriate forms of CDS.^5^^,^^6^^,^^63^

A key strength of this study is that it involved a comprehensive, consultative, and continuous improvement development process that was informed by evidence, and local and international multidisciplinary expert opinion. There were some limitations associated with the literature search to identify relevant articles. The literature search was not systematic, and some relevant articles might have been missed. However, this was mitigated by consulting with experts to ensure that important content related to CDS design was covered by the guideline. The number of experts consulted during phase 1 was limited to a small number of local experts, and phase 2 recruitment was somewhat spontaneous. Although part of the guideline was evaluated through a case study based workshop, this was time limited, and some groups did not have sufficient time for discussion. When applied in practice, CDS decision makers should allow sufficient time for the guideline’s application, potentially over multiple, iterative sessions. However, lack of time to fully apply the guideline may be a challenge in time-constrained real-world settings. The study also did not evaluate the guideline using quantitative metrics.

## Conclusion

With the increasing application of HF and systems safety thinking to HIT design, there is a significant opportunity for best practice design principles to guide design decisions about CDS and ensure they are appropriate. By drawing on a wide range of sources, a 2-phased development process culminated in the development of a comprehensive, accessible, and current HF-informed best practice design guideline, tailored to the needs of the CDS context. The guideline is now freely accessible across all public hospitals and statewide organizations within our state. Overall, this study found that the developed CDS guideline met expectations around CDS needs, was considered useful, and generated significant interest in further access to and application of the guideline. This underscores the guideline’s potential as a critical tool in ensuring safe and effective CDS.

## Potential Competing Interests

Selvana Awad was supported by an Australian Government Research Training Program (RTP) Scholarship. The other authors report no conflicts of interest.

## Ethics Statement

This project was determined to be a quality improvement study, and ethics approval was not required.

## Supplemental Online Material

Appendices
