# Supplementary material for: Development of a Human Factors–Based Guideline to Support the Design, Evaluation, and Continuous Improvement of Clinical Decision Support
Source: Mayo Clin Proc Digit Health. 2024 Nov 27;3(1):100182. doi: 10.1016/j.mcpdig.2024.11.003 (PMC11975816; doi:10.1016/j.mcpdig.2024.11.003)
Supplement: Appendices [file mmc1.pdf]

Appendix 1 – Additional information on the search strategy

Search terms associated with searched concepts

**Concept**	**Examples of keywords used**
Concept 1: Human factors design	ergonomic<br>human factors<br>human factors engineering<br>usability<br>user-centered / user-centered<br>human-centered / human centered<br>design<br>heuristic
Concept 2: Clinical decision support	Clinical decision support<br>Electronic medical record / electronic health record<br>Electronic medication management system<br>Computerized / Computerized Provider Order Entry<br>Medical records system

- Search terms were inputted into PubMed and titles and abstracts published from 2012 were screened by 1 researcher
- Medical informatics and human factors journals were also individually searched for relevant papers using appropriate search terms for these journals. Examples included:
  - BMJ Health & Care Informatics
  - International Journal of Medical Informatics
  - JMIR Medical Informatics
  - Journal of the American Medical Informatics Association
  - Human Factors
  - Applied Ergonomics, and Ergonomics

Appendix 2 - Industry standards and literature used to inform sections of the CDS guideline

**Industry Standards**
International Electrotechnical Commission (IEC) Technical Standards - IEC 62682 – Management of Alarm Systems for Process Industries[36]
American National Standards Institute (ANSI)/International Society of Automation (ISA)- ANSI/ISA-18.2-2016 - Management of Alarm Systems for the Process Industries (commonly referred to as ISA-18.2)[37]
American National Standards Institute (ANSI)/Association for the Advancement of Medical Instrumentation (AAMI) HE75:2009 - Human factors engineering – Design of medical devices[36]
The Engineering Equipment and Materials Users Association Alarm Systems Guide to design, management and procurement Publication 191[53]
National Institute of Standards and Technology (NIST) - NIST.GCR 15-996 Technical Basis for User Interface Design of Health IT[48]
Human Factors Design Standard (DOT/FAA/HF-STD-001B)[40]
Human Factors Design Standard (HF-STD-001)[49]
ISO 9241-210 Ergonomics of human-system interaction - Part 210: Human-centred design for interactive systems[9]
ISO 9241-125:2017(E) Ergonomics of human-system interaction Part 125: Guidance on visual presentation of information[50]
ISO 9241-143:2012(E) Ergonomics of human-system interaction Part 143: Forms[51]
MIL-STD-147G Department of Defense Design Criteria Standard Human Engineering[52]
**Academic literature**
Journal papers

Appendix 3a – Flowchart tested which guides the appropriate consideration of CDS and options

Step	Guiding Questions / Considerations	Tools / Resources
**Problem Definition**	• What is the **clinical risk** / **business need** / **problem** / **requirement** / **use case**?<br>• What **clinical practice / workflow** / **behaviour** are we trying to support or change? What **errors** are we trying to prevent?<br>• What are the **causes** / **contributing factors of the problem you are trying to solve**?<br>• What clinical outcome are we seeking to achieve?	Use design thinking and safety analysis techniques to explore the problem / risk & contributing factors.
**Appropriateness**	• **Effectiveness:** Will CDS be effective as a risk control / intervention? Will use of CDS result in better outcomes than reliance on clinical judgement alone? What is the evidence?<br>• **Benefits:** What is the potential benefit of the CDS to patients, clinicians, and other staff?<br>• **Unintended consequences:** Are there unintended consequences of the CDS to patients, clinicians, and other staff?<br>• **Clinical feasibility:** What is the clinical feasibility of implementing the proposed CDS? Is there a lack of clinical consensus around the clinical area / practice? Are there too many nuanced variables impacting the clinical decision that cannot be feasibly reflected in system functionality?<br>• **Usability and Human Factors:** Assessing the potential impact of the CDS system on usability and its alignment with intended workflows in the specific clinical setting. Consideration should be given to whether the system will enhance or hinder usability, and whether it may introduce distractions or interruptions in the clinical workflow.<br>• **Other interventions:** Are other interventions outside of the clinical information system more appropriate? (e.g., training, change management, clinical processes)<br>• **Existing/New CDS:** consideration to existing/planned CDS that may be complimentary or conflicting to new CDS.	Use hazard and safety analysis approaches to understand the appropriateness of CDS as a risk control / intervention.<br><br>Review literature to understand the evidence for CDS in this context.<br><br>Review available clinical guidelines and consult with relevant clinical groups to assess clinical feasibility.
**Prioritization**	• **Risk Assessment:** What is the associated risk of not implementing the CDS? Consider severity and likelihood.<br>• **Priority / Importance:** Considering the broader clinical information system, what is the relative priority/importance of the proposed CDS?	Use a risk matrix to quantify risk.<br><br>Identify other CDS (e.g., other alert triggers and colors) in the workflow to understand relative priority within the system.

Is CDS considered appropriate, clinically feasible and of sufficient priority to implement?

YES → Consider appropriate and technically feasible CDS option/s

AND

NO → Consider other mitigations / interventions / risk controls outside of the clinical information system

Examples: training, change management, and

Step	Guiding Questions / Considerations	Tools / Resources
**CDS Options:**<br><br>**Passive and/or Interruptive**	Consider the following to guide identification of appropriate CDS options:<br><br>• **Workflow integration:** What point within the workflow/task/process is the CDS required to support decision making? Who is the information targeted at? It may be appropriate to consider CDS combinations. *CDS should present the right information, at the right time, to the right people who need to be aware of the information and/or take action.*<br>• **Risk assessment:** What is the level of risk? Consider severity and likelihood. *Interruptive options should be reserved for high-risk scenarios requiring the user to stop before proceeding further. Some high-risk scenarios may be adequately supported by passive options. In some cases, using a combination of passive and interruptive CDS options may be appropriate.*<br>• **Workflow impact:** What is the impact on workflow and what other CDS appears / triggers within the workflow?<br>• **Technical feasibility:** what is technically possible? What is the build and maintenance burden?<br>• **Additional mitigations / controls:** In addition to using CDS, is there a need for additional mitigations / risk controls outside of the clinical information system?<br>• **Lessons Learnt:** Engaging with external teams using the same product to gather insights and lessons that can inform the design of the clinical decision support (CDS) system.	Map out the workflow/process/task and identify target users.<br><br>Use a risk matrix to quantify risk.<br><br>Identify other CDS (e.g., other alert triggers and colors) within the workflow.<br><br>Use data to simulate potential volume and frequency of CDS displaying / triggering / alerting.<br><br>Use hazard and safety analysis approaches to understand the need for additional mitigations / risk controls.

Appendix 3b – Table tested which lists passive and interruptive CDS options, and use cases

Classification		Modalities	Purpose	Examples of Use Cases
**Passive**	Information display and interface design	• Colour, size, white space, contrast, dynamic states (changing states)<br><br>• Typographic design: bold, caps, italics, underline, strikethrough.	Increase the salience of critical information or actions within the design of a user interface	• Specific colours for out-of-range results e.g. red for results that are critically high<br>• Highlighting critical information e.g. using a red colour to indicate urgent alerts or warnings in a patient monitoring system.<br>• Dynamic states (changing states) e.g. animating a progress bar or loading indicator to provide feedback and reassure users that an action is in progress.
		• Graphical displays and trending<br>• Information grouping and summaries	Improve the recognisability of trends, relationships, and out-of-range results<br><br>Support the cognitive process for key clinical decisions by group	• Between the Flags displays (visual markers to indicate whether a patient's observations are within or out of range)<br>• Graphs that overlay blood glucose levels and medication doses<br>• Summary pages for specific tasks e.g. workflow pages that summarise relevant information for specific conditions or tasks<br>• Data reports and dashboards showing patients on restricted antimicrobials requiring review
		Presentation of contextually relevant reference information at appropriate times in workflows	Improve access to contextually relevant and advisory information to support decision making during task completion	• Displaying relevant lab results and reference ranges during particular tasks e.g. display blood glucose levels when prescribing diabetes medications<br>• Displaying relevant clinical information via reference text e.g. displaying guidance on venous thromboembolism (VTE) risk factors during VTE risk assessment

				• Displaying relevant patient information e.g. patient weight during medication prescribing
	Functionalities to support task completion	Non-interruptive reminders and notifications	Notify the user of non-urgent information/ tasks	• Notifications which can be attended to at the convenience of the clinician that appear on a task list
		Defaults and templates	Enhance efficiency, reduce effort and guide the user to enter required information or perform required tasks<br><br>Reduces the risk of transcription errors<br><br>Improve likelihood of making appropriate selection	• Appropriate defaults for order entry e.g. medication order sentences with pre-populated doses and frequencies<br>• Including only appropriate routes on a drop-down list<br>• Changing the default medication option to the most appropriate medication<br>• Removing inappropriate options to reduce chance of selection errors<br>• Documentation templates with pre-populated headings and information<br>• Ability to select available results or values to populate a note or discharge summary
	Functionalities to support diagnosis and management	Diagnostic support	Enhance diagnosis and assessment processes<br><br>Reduce effort and cognitive load by presenting relevant information to the clinician to support their decision making<br><br>Improve likelihood of making appropriate decisions	• Standardised assessments e.g. risk screening tools<br>• Algorithms that flag at-risk patients via dashboards or similar e.g. patients at risk of deterioration<br>• Situations where diagnostic decisions need to be made under time pressure and in chaotic environments<br>• Final step of clinical decision-making (e.g., the system provides possible diagnoses based on all assessment data).<br>• Algorithm that flags tests that may support diagnosing a patient based on a set of conditions being met (recommendations for tests may be displayed on a workflow page or other appropriate functionality)

		Order sets and other functionalities to support management	Guide the management of conditions to ensure patients receive appropriate tests, orders and care<br><br>Reduce effort and save clinicians' time by allowing groups of orders to be placed at one time<br><br>Reduce cognitive load by presenting relevant information to the clinician to support their decision making<br><br>Improve likelihood of making appropriate decisions	• Order set with groups of orders based on clinical guidelines. These groups of orders can include medications, pathology, imaging, and patient care tasks, e.g. order set for the management of community-acquired pneumonia.<br>• Algorithm that flags orders that may benefit a patient based on a set of conditions being met (recommendations for orders may be displayed on a workflow page or other appropriate functionality)<br>• Providing the ability to pre-select orders or link orders so that if one order is selected, other appropriate orders are selected, or if an order is selected, it will not allow selection of other orders that may be inappropriate/contraindicated (e.g. sepsis selection of diagnostic test or antibiotic order combinations)
		Pathways and other functionalities that include decision support for diagnosis and management<br><br>NB. A pathway is a structured and standardised approach or set of guidelines that outlines the recommended steps and actions to assess and manage patients	Guide the diagnosis (including appropriate tests) and management of conditions (including appropriate orders)<br><br>Reduce effort and save clinicians' time by allowing groups of orders to be	• Pathway for assessing and managing patients with various conditions e.g. acute coronary syndrome, VTE risk<br>• Tools that identify at risk patients and recommend management options

		with a specific medical condition or risk.	placed at one time<br><br>Reduce cognitive load by presenting relevant information to the clinician to support their decision making<br><br>Improve likelihood of making appropriate decisions	
**Interruptive**	Functionalities that prompt actioning of urgent tasks and alerting of safety-critical events/information requiring immediate action or behaviour change due to a set of conditions being met	Soft stop alerts	Alerts that pop up and prompt the user to 'stop and think' before proceeding. They may include warning statements requiring immediate awareness. The user is able to proceed after actively acknowledging the alert.	• Alerts that appear within the workflow containing safety-relevant information for the clinical decision being made e.g. duplicate therapy alerts where the user is prompted to think about the appropriateness of the duplicate therapy (noting in some cases, it may be clinically appropriate).<br>• Alerts that prompt the user to stop and consider actions based on a set of conditions being met that would otherwise have not been considered e.g. ordering a specific test in a given clinical setting where another may be more clinically appropriate or another test should be added (these alerts prompt a change in behaviour as a result of being presented with the information in the alert)
		Hard stop alerts	Alerts that pop up and prevent the user from proceeding until the required action is undertaken.	• Alerts that appear within the workflow requiring immediate action due to a high risk of harm if the required action is not undertaken e.g. the user is unable to proceed until they de-select a medication order

				to which the patient has a documented severe allergy.
		Other forcing functions	These may be other functionalities which prevent the user from proceeding until the required action is undertaken.	• An admission order set cannot be signed off and the user is unable to proceed until a safety critical component has been completed.<br>• The system does not allow the user to enter a figure for a specific data element (e.g. weight) in a form beyond an upper limit.

Appendix 4 – Scenarios used for tested during Phase 2

<u>Scenario #1: Blood Product Management</u>

An EMR system is utilised to ensure the safe and appropriate use of blood products. However, there have been cases of incorrect blood product selection and administration, potentially jeopardising patient safety.

Clinicians reported:

- the EMR system's interface lacked clear visual cues or prompts to guide them through the selection process and ordering workflow.
- the ordering workflow is complex and includes multiple steps and navigation to a number of different screens.
- relevant patient data, such as transfusion history or specific contraindications, may be invisible until digging through multiple tabs or hidden within lengthy electronic health records. This results in them unintentionally choosing the wrong blood product without noticing.
- blood products are similarly labelled and not easy to distinguish from one another

<u>Scenario #2: Paediatric Medication Prescription</u>

An EMR system is utilised for medication prescribing to ensure accurate dosing and prevent adverse drug events. However, there have been cases of incorrect dosage calculations associated with medication prescribing, particularly in paediatric patients.

Clinicians have reported difficulties in calculating and determining appropriate medication dosages for paediatric patients within the EMR system. They reported the system and interface:

- isn’t tailored to paediatric dosing lacking specific considerations towards age, weight, diagnosis, and other factors, making it hard to calculate accurately and hard to select appropriate medication
- does not provide any guidance on appropriate dosages based on age and other factors
- requires navigation to different screens to gather the required information such as weight for dose calculations
- does not include functionality to support more automated dose calculation i.e. doses need to be calculated manually and inputted into a field

Appendix 5. Section of the CDS guideline and key content

	Section	Key content
**1**	About this document	• Document rationale, purpose and scope<br>• Guidance on how to use the document
**2**	Determining the appropriateness of CDS and potential options	• Flowchart to guide whether CDS is appropriate<br>• Table with potential CDS options<br>• Guidance on the process for designing, evaluating and continuously improving CDS
**3**	CDS governance and review	• Guidance on CDS governance and review including personnel/subject matter experts who should be involved in decision-making about CDS
**4**	Specific design guidance on CDS options	• Specific design guidance on CDS options including design principles and additional considerations relevant to each option

Appendix 6 - Industry standards and literature used to inform sections of the CDS guideline

Source	Sections informed by references	Example of content that was drawn from the source
**Industry Standards**		
International Electrotechnical Commission (IEC) Technical Standards - IEC 62682 – Management of Alarm Systems for Process Industries[36]	1,2,4	Principles and processes for managing alerts.
American National Standards Institute (ANSI)/International Society of Automation (ISA)-ANSI/ISA-18.2-2016 - Management of Alarm Systems for the Process Industries (commonly referred to as ISA-18.2)[37]	1,2,4	The need for a strategy or philosophy for managing alerting.
American National Standards Institute (ANSI)/Association for the Advancement of Medical Instrumentation (AAMI) HE75:2009 - Human factors engineering – Design of medical devices[36]	1, 2, 3, 4	Design teams should seek input from a wide variety of users who are appropriately representative of typical intended users.
The Engineering Equipment and Materials Users Association Alarm Systems Guide to design, management and procurement Publication 191[53]	1, 2, 3, 4	The purpose of an alert is to direct the user's attention to safety-critical information requiring timely attention or actioning.
National Institute of Standards and Technology (NIST) - NIST.GCR 15-996 Technical Basis for User Interface Design of Health IT[48]	1,2,4	Do not rely on color alone to draw attention to critical information.<br><br>Limit alerts to those that are clinically essential to ensure safe and effective patient care.
Human Factors Design Standard (DOT/FAA/HF-STD-001B)[40]	1,2,4	One meaning per color: Each color should represent only one category of displayed data.
Human Factors Design Standard (HF-STD-001)[49]	1,2,4	Where data entry is required to support auto-population (e.g. to enable system calculation of a dose), the system should prompt the user to correctly enter

		required data. The system should provide a means to indicate to the user that data are incomplete, missing, unreliable or invalid.
ISO 9241-210 Ergonomics of human-system interaction - Part 210: Human-centred design for interactive systems[9]	1, 2, 3, 4	Ensuring that the design is informed by end-user feedback and the design process is iterative.
ISO 9241-125:2017(E) Ergonomics of human-system interaction Part 125: Guidance on visual presentation of information[50]	1, 2, 4	Color assignments should be used in accordance with cultural conventions.
ISO 9241-143:2012(E) Ergonomics of human-system interaction Part 143: Forms[51]	1, 2, 4	Fields should contain default values whenever possible and appropriate for the task. If default values are likely to vary across users but remain relatively consistent across the tasks of a specific user, methods should be provided to allow the user to customize default values.
MIL-STD-147G Department of Defense Design Criteria Standard Human Engineering[52]	1, 2, 4	Capital letters should be used for signal words e.g. DANGER, CAUTION, ATTENTION, NOTICE
**Academic Literature**		
Journal papers	1, 2, 3, 4	The need for CDS to integrate with the workflow. It is about presenting the right information to the right person, at the right time.
